# Distinguishing the Signals of Gingivitis and Periodontitis in Supragingival Plaque: a Cross-Sectional Cohort Study in Malawi

**DOI:** 10.1128/AEM.01756-16

**Published:** 2016-09-16

**Authors:** Liam Shaw, Ulla Harjunmaa, Ronan Doyle, Simeon Mulewa, Davie Charlie, Ken Maleta, Robin Callard, A. Sarah Walker, Francois Balloux, Per Ashorn, Nigel Klein

**Affiliations:** a Institute for Child Health, UCL, London, United Kingdom; b Centre for Mathematics and Physics in the Life Sciences and Experimental Biology, UCL, London, United Kingdom; c Center for Child Health Research, University of Tampere and Tampere University Hospital, Tampere, Finland; d University of Malawi College of Medicine, Blantyre, Malawi; e MRC Clinical Trials Unit at UCL, London, United Kingdom; f UCL Genetics Institute, UCL, London, United Kingdom; g Department of Paediatrics, Tampere University Hospital, Tampere, Finland; Stanford University

## Abstract

Periodontal disease ranges from gingival inflammation (gingivitis) to the inflammation and loss of tooth-supporting tissues (periodontitis). Previous research has focused mainly on subgingival plaque, but supragingival plaque composition is also known to be associated with disease. Quantitative modeling of bacterial abundances across the natural range of periodontal severities can distinguish which features of disease are associated with particular changes in composition. We assessed a cross-sectional cohort of 962 Malawian women for periodontal disease and used 16S rRNA gene amplicon sequencing (V5 to V7 region) to characterize the bacterial compositions of supragingival plaque samples. Associations between bacterial relative abundances and gingivitis/periodontitis were investigated by using negative binomial models, adjusting for epidemiological factors. We also examined bacterial cooccurrence networks to assess community structure. The main differences in supragingival plaque compositions were associated more with gingivitis than periodontitis, including higher bacterial diversity and a greater abundance of particular species. However, even after controlling for gingivitis, the presence of subgingival periodontitis was associated with an altered supragingival plaque. A small number of species were associated with periodontitis but not gingivitis, including members of Prevotella, Treponema, and Selenomonas, supporting a more complex disease model than a linear progression following gingivitis. Cooccurrence networks of periodontitis-associated taxa clustered according to periodontitis across all gingivitis severities. Species including Filifactor alocis and Fusobacterium nucleatum were central to this network, which supports their role in the coaggregation of periodontal biofilms during disease progression. Our findings confirm that periodontitis cannot be considered simply an advanced stage of gingivitis even when only considering supragingival plaque.

**IMPORTANCE** Periodontal disease is a major public health problem associated with oral bacteria. While earlier studies focused on a small number of periodontal pathogens, it is now accepted that the whole bacterial community may be important. However, previous high-throughput marker gene sequencing studies of supragingival plaque have largely focused on high-income populations with good oral hygiene without including a range of periodontal disease severities. Our study includes a large number of low-income participants with poor oral hygiene and a wide range of severities, and we were therefore able to quantitatively model bacterial abundances as functions of both gingivitis and periodontitis. A signal associated with periodontitis remains after controlling for gingivitis severity, which supports the concept that, even when only considering supragingival plaque, periodontitis is not simply an advanced stage of gingivitis. This suggests the future possibility of diagnosing periodontitis based on bacterial occurrences in supragingival plaque.

## INTRODUCTION

Periodontal disease is a major public health problem, particularly in low-income settings like sub-Saharan Africa (1). Aside from irreversible tooth loss, chronic periodontitis may also increase the risk of adverse systemic conditions (2), such as cardiovascular disease (3) and preterm birth; however for preterm birth, different studies have reported conflicting results (4). The association between periodontitis and systemic disease may be due to both increased systemic inflammation and to translocation of bacteria into the bloodstream (5). Despite its importance, the microbial ecology of periodontal disease in different oral habitats remains incompletely understood. Studies of the oral microbiome in periodontal disease typically focus on small populations in developed countries with advanced dental health care systems, which may not be representative of the natural history of periodontal disease in the absence of treatment (6).

In periodontal disease, the immune system responds with inflammation to oral biofilms (7). After an initial focus on identifying particular periodontal pathogens (8), it is now widely accepted that oral bacterial communities undergo a shift or dysbiosis (9) and that the presence of particular disease-associated species may exacerbate the inflammatory reaction to commensal bacteria (10). The two main features of periodontal disease are gingival inflammation (gingivitis) and the formation of periodontal pockets (periodontitis). While it is clear that gingivitis always precedes periodontitis (11), gingivitis does not always progress to periodontitis (12), suggesting that these conditions may not simply represent different stages of a continuous spectrum of disease. While there is some evidence that a steady continuous progression may be expected (13), most models involve acute bursts of exacerbation and longer periods of remission (14, 15).

Despite this knowledge, studies of oral bacteria in periodontal disease often fail to capture the full range of periodontal conditions, from health through gingivitis to periodontitis. In supragingival plaque, in particular, comparing only healthy subjects with subjects suffering from periodontitis may lead to bacterial associations being attributed to periodontitis alone, despite the fact that they may also be present in subjects with gingivitis. To explain the progression of disease and identify factors uniquely attributable to periodontitis, it is necessary to compare subjects across the full range of periodontal severities. In itself, this is not a novel concept, with many previous studies investigating bacterial associations with disease using checkerboard DNA-DNA hybridization (16–18). Earlier studies were targeted at a small number of bacterial species (typically around 40). The advent of high-throughput 16S rRNA gene amplicon sequencing has facilitated the improved analysis of the total bacterial diversity in the oral cavity (19, 20), identifying around 1,000 species that may be present (10) and showing that samples from the mouth typically have higher alpha diversities than those from other body sites (21, 22). Recent studies have used such amplicon sequencing to characterize subgingival plaque across a range of periodontal conditions, finding differences between subjects with gingivitis and periodontitis (23, 24). Work on supragingival plaque has been less common due to the fact that it does not have a direct link to inflammation and the subsequent loss of attachment in periodontitis. It therefore remains ambiguous whether, for supragingival plaque, periodontitis can be simply considered an advanced stage of gingivitis or if there are detectable differences in bacterial composition.

To address this question, we investigated bacterial abundances in supragingival plaque using quantitative modeling that takes into account gingivitis (quantified by bleeding on probing [BoP]) and periodontitis (quantified by periodontal pocket depth) in a cross-sectional cohort of 962 Malawian women who had recently given birth (25).

We used negative binomial models that were originally developed for transcriptome sequencing (RNA-seq) experiments (26), making use of absolute (i.e., unnormalized) read counts to avoid losing information—a downside of other statistical approaches applied to marker gene data like rarefying (27). After fitting a negative binomial distribution to the count data for a given species, the mean of this distribution was then used as the output of a generalized linear model with a logarithmic link using experimental variables (e.g., disease severity) as inputs, which allowed the identification of differentially abundant species. This approach considers bacterial species to be independent, but in reality, oral bacteria exist in complex polymicrobial biofilms (28). Therefore, we also applied a cooccurrence analysis to periodontitis-associated bacteria to identify important members of the community.

In summary, we aimed to identify the effects of periodontitis on supragingival plaque after controlling for gingivitis severity, separating and distinguishing the signals of these two features of periodontal disease.

## MATERIALS AND METHODS

### Study population.

Women who were analyzed in this study were participants in the iLiNS-DYAD-M trial (International Lipid-Based Nutrient Supplements study group, enrolling mother-child dyads in Malawi; ClinicalTrials registration no. NCT01239693) (25). This was a randomized controlled trial that investigated the effects of the following three nutritional supplements on birth outcomes: lipid-based nutrient supplement (LNS), multiple micronutrients (MMN), or iron-folic acid (IFA). Women were eligible for enrollment in the trial if they were pregnant for <20 weeks, >14 years old, had no chronic illnesses requiring frequent medical care, had no allergies, had no evident pregnancy complications (edema, blood hemoglobin of <50 g/liter, systolic blood pressure of >160 mm Hg, or diastolic blood pressure of >100 mm Hg), no earlier participation in the same trial, and no concurrent participation in any other trail.

A total of 1,391 pregnant women were enrolled between February 2011 and August 2012 at antenatal clinics at two hospitals (Mangochi and Malindi) and two health centers (Lungwena and Namwera) in Mangochi district, Malawi. All women were self-reported nonsmokers and were given two courses of preventive malaria treatment with sulfadoxine-pyrimethamine (SP; three tablets of 500 mg sulfadoxine and 25 mg pyrimethamine orally), one at enrollment and one between the 28th and 34th gestational week. After giving birth, 1,229 women completed an oral health examination, consisting of a clinical examination and a panoramic X-ray of the jaws. A total of 1,024 women had this examination within 6 weeks of delivery of a single infant (mothers of twins were excluded) and were included in further analysis. After excluding women without a supragingival sample (*n =* 59) and those with an unknown HIV status (*n* = 3), 962 women remained for our cross-sectional analysis.

### Classification of periodontal disease.

Gingivitis was measured by the number of dental arch sextants with bleeding on probing (BoP) out of six, with three sextants on each jaw (left, middle, and right). For periodontitis classification, each tooth was examined for evidence of deepened dental pockets, both clinically and radiologically. A tooth was defined as having periodontitis if either a ≥4-mm pocket was measured in clinical examination or a vertical bony pocket was identified at least at the cervical root level radiologically. A woman was defined as having periodontitis if she had at least three teeth with periodontitis or at least one dental arch sextant with horizontal bone loss (at least at the cervical level). The examination and classification methods are explained in detail elsewhere (29).

### Sample collection.

Supragingival dental plaque samples were collected by swabbing the gingival margin of each tooth with a sterile plastic swab stick with a nylon fiber tip (microRheologics no. 552; Coban, Brescia, Italy). After transfer in a cold box with ice packs to a laboratory, swabs were stored in cryovials at −20°C before being transferred to −80°C.

### DNA extraction and sequencing.

We used Illumina compatible primers (785F, GGATTAGATACCCBRGTAGTC, and 1175R, ACGTCRTCCCCDCCTTCCTC) (30) that amplify the V5 to V7 region of the 16S rRNA gene to generate a sequencing library (31). Each sample was amplified with dual indexes on the forward and reverse primer. All barcodes and adapter sequences used have been previously published (32). Each reaction mixture was set up with 1× Molzym PCR buffer (Molzym), 200 μM deoxynucleoside triphosphates (dNTPs) (Bioline), 0.4 μM forward and reverse primer with barcode attached, 0.025 μM MolTaq (Molzym), and 5 μl of template DNA and PCR grade water (Bioline) to make a final reaction mixture volume of 25 μl. Cycling parameters were as follows: 94°C for 3 min, 30 cycles of 94°C for 30 s, 60°C for 40 s, and 72°C for 90 s, and one final extension at 72°C for 10 min.

Samples were purified and pooled into an equimolar solution using the SequalPrep normalization plate kit (Life Technologies) and further cleaned using AMPure XP beads (Beckman Coulter), both per the manufacturer's recommendations. After quantification using Qubit 2.0 (Life Technologies), the library was diluted and loaded into the MiSeq reagent cartridge at 10 pM. MiSeq runs were set to generate 250-bp paired-end reads and two 12-bp index reads for each sample.

### Taxonomic classification.

Sequenced reads were merged, demultiplexed, and quality filtered (minimum average Phred score of >25) using QIIME v1.8.0 (33). Closed-reference operational taxonomic units (OTUs) were picked at a 98.5% similarity against the Human Oral Microbiome Database (HOMD) v13.2 (20) using USEARCH v6.1.544 (34) in QIIME v1.8.0 (33) with parallel_pick_otus_usearch61_ref.py. We used a 98.5% sequence similarity because this is the threshold used to define taxa in HOMD, as it approximately corresponds to species-level clusters for most oral bacteria (20). This approach identified 664 bacterial OTUs corresponding to 13,049,932 reads. The mean number of reads per sample was 13,565 ± 6,833.

Closed-reference OTU picking suffers from a number of issues, including sensitivity to the order of reference sequences when sequences are identical over the region considered (35). This is a particular problem when sequences are similar; there exist oral bacteria that have >99% sequence similarity in given regions of the 16S rRNA gene but occupy separate oral habitats (36). For this reason, we also performed minimum entropy decomposition (MED) on reads. MED is an unsupervised version of the oligotyping pipeline (37), which allows a greater resolution of microbial diversity by partitioning sequences based on sites with high positional entropy in a reference-free manner (36).

After merging overlapping reads, the average sequence length was 369 bases. We filtered sequences with an expected error of more than 1 using fastq_filter in VSEARCH v1.11.1 (38). We then discarded all sequences that were shorter than 350 bases or longer than 380 bases but performed no other quality filtering (e.g., length truncation) because MED assumes that length variation is biologically meaningful. We ran MED v2.1 on 14,449,794 sequences (information on the reads discarded at each stage is available in the supplemental material). Because we wanted to be able to detect rare sequences, we set the minimum substantive abundance parameter (*M*) to 1,444 (0.1% of the total number of reads) and the maximum variation allowed within a node (*V*) to 3. All other parameters were set to their default values. We assigned taxonomy to MED phylotypes using the Global Assignment of Sequence Taxonomy (GAST) (39) with VSEARCH v1.11.1 replacing USEARCH.

### Statistical analyses. (i) Diversity.

We fitted a multivariate linear regression model to predict species richness (observed number of species) and the Shannon index (a measure of richness and evenness) using gingivitis, periodontitis, and the variables listed in Table 2 for 811/962 samples with complete data and >5,000 reads. Richness and Shannon index were averaged over 100 iterations of rarefying to 5,000 reads per sample. Backwards stepwise reduction by the Akaike information criterion (AIC) (40) was used to select the final model.

### (ii) Differential abundances.

We used DESeq2 v1.6.3 (26) in Phyloseq to model abundances. DESeq2 uses negative binomial generalized linear models to compare the absolute number of reads for each taxon between categories (27). Gingivitis was included as a continuous variable (BoP ranging from 0 to 6) and periodontitis as a binary factor. The model also contained terms controlling for potential confounders (study site, nutritional intervention, HIV status, and sequencing run). *P* values were corrected for multiple testing using the Benjamini-Hochberg procedure (41). Full DESeq results for gingivitis and periodontitis are available in the supplemental material (Data Set S1).

### (iii) Correlation networks.

To facilitate a higher resolution of the network of periodontitis-associated bacteria, we selected all MED phylotypes that had representative sequences with >98.5% sequence similarity to periodontitis-associated HOMD OTUs. We calculated pairwise Spearman correlation coefficients between these MED phylotypes across samples. We used the SparCC procedure to estimate correlations from compositional data using log-ratio transformed abundances (42) with default parameters (20 inference iterations and a correlation strength exclusion threshold of 0.1). To calculate pseudo *P* values (two-sided *t* test), we shuffled the data sets for each group 100 times and repeated the procedure, removing correlations that were not significant (*P* < 0.05, no multiple testing correction). Networks of strong correlations, defined as being outside of the 95% confidence interval (CI) for the mean correlation between nodes (mean + 1.96 × standard deviation [SD], e.g., 0.405 for the network in Fig. 4a) were visualized as networks with qgraph v1.3.1 (43) using the Fruchterman-Reingold algorithm for node placement (44).

### Accession number(s).

Reads were deposited in the European Nucleotide Archive under study accession no. PRJEB15035 (see the supplemental material for details).

## RESULTS

### Description of cohort.

A total of 962 Malawian women were included in our analysis, with a mean age of 25.4 ± 6.2 years. Of these women, 140 (14.6%) had no periodontal disease, 822 (85.4%) had gingivitis (bleeding on probing [BoP] score of ≥1), and 307 (32.0%) had periodontitis (Table 1). Gingivitis and periodontitis were significantly correlated (Spearman's ρ = 0.44), with the majority of women with periodontitis having high levels of gingivitis. Periodontitis and gingivitis were more common in women who were older, had lower socioeconomic status, and had fewer years of education (Table 2; for modeling, see Table S1 in the supplemental material).

**TABLE 1:** Breakdown of all women by severity of periodontal disease

Periodontitis	No. of women according to severity of periodontal disease (no. dental arch sextants with BoP)						
	0	1	2	3	4	5	6
No	137	72	95	111	72	63	102
Yes	4	11	23	27	51	50	145

**TABLE 2:** Demographic characteristics broken down by severity of periodontal disease

BoP (no.)	Periodontitis	Total no. of women	Age (yr [SD])	No. with positive HIV test (%)	No. with malaria (%)^*a*^	Mean BMI (SD)	Mean education (yr [SD])	No. with anemia (%)^*b*^	Socio-economic status (SD)^*c*^	No. of women from each site^*d*^	No. of women receiving each nutritional intervention^*e*^	No of samples on each sequencing run^*f*^
0	No	140	23.4 (5.8)	27 (19.3)	37 (26.6)	22.7 (3.2)	5.6 (3.6)	36 (25.7)	0.38 (1.22)	36/37/18/49	43/53/44	47/49/41/3
1	No	72	23.9 (5.9)	7 (9.7)	16 (22.2)	22.6 (3.4)	5.1 (3.8)	12 (16.7)	0.19 (1.11)	25/9/17/21	32/19/21	34/26/12/0
	Yes	11	31.6 (6.1)	1 (9.1)	1 (9.1)	22.7 (2.4)	4.4 (3.3)	3 (27.3)	−0.35 (0.62)	6/2/1/2	8/0/3	3/5/3/0
2	No	95	24.7 (6.2)	11 (11.6)	22 (23.2)	22.1 (2.6)	4.4 (3.6)	19 (20.0)	0.10 (1.10)	39/19/13/24	38/34/23	31/41/23/0
	Yes	23	27.5 (6.2)	5 (21.7)	5 (21.7)	21.7 (2.0)	2.7 (3.3)	4 (17.4)	−0.16 (0.91)	13/1/4/5	5/11/7	9/7/7/0
3	No	111	24.4 (5.4)	11 (9.9)	32 (28.8)	21.7 (2.3)	4.3 (3.3)	21 (18.9%)	−0.12 (0.84)	41/22/22/26	40/34/37	36/34/39/2
	Yes	27	26.5 (5.7)	4 (14.8)	3 (11.1)	22.2 (2.7)	3.6 (3.0)	6 (22.2)	−0.20 (0.91)	11/6/3/7	11/4/12	11/6/10/0
4	No	72	25.0 (6.4)	9 (12.5)	16 (22.2)	21.7 (2.2)	3.4 (3.0)	11 (15.3)	−0.16 (0.80)	28/16/10/18	16/26/30	26/28/18/0
	Yes	51	26.9 (5.4)	8 (15.7)	11 (21.6)	21.8 (2.7)	3.3 (3.1)	7 (13.7)	−0.17 (0.81)	27/3/7/14	14/19/18	23/7/21/0
5	No	63	24.9 (5.2)	7 (11.1)	12 (19.0)	21.6 (2.4)	4.0 (3.6)	15 (23.8)	−0.16 (0.81)	22/11/9/21	22/23/18	26/13/24/0
	Yes	50	26.6 (5.9)	5 (10.0)	7 (14.0)	21.8 (3.1)	2.4 (2.8)	5 (10.0)	−0.36 (0.61)	18/11/7/14	16/15/19	21/12/17/0
6	No	102	24.5 (5.5)	10 (9.8)	18 (17)	21.9 (2.3)	3.5 (3.0)	26 (25.7)	−0.20 (0.81)	36/24/16/26	33/41/28	18/46/36/2
	Yes	145	28.3 (7.0)	30 (20.7)	28 (19.3)	22.1 (2.5)	2.9 (3.0)	32 (22.1)	−0.27 (0.74)	66/28/17/34	45/48/52	59/43/41/2

a Malaria was diagnosed with a rapid diagnostic test obtained from a finger prick.

b Anemia was defined as a hemoglobin count of <110 g/liter.

c A proxy for socioeconomic status was created from a principal components analysis by combining information on the building material of the house, main source of water and electricity, sanitary facilities, and main type of cooking fuel used.

d Women were enrolled at the following four sites: Lungwena, Malindi, Namwera, and Mangochi, respectively.

e Women received one of the following three nutritional interventions: IFA, MMN, or LNS, respectively.

f Supragingival samples were run on one of four sequencing runs on Illumina MiSeq.

### Plaque richness and diversity are higher in more severe gingivitis and periodontitis.

**FIG 1:**
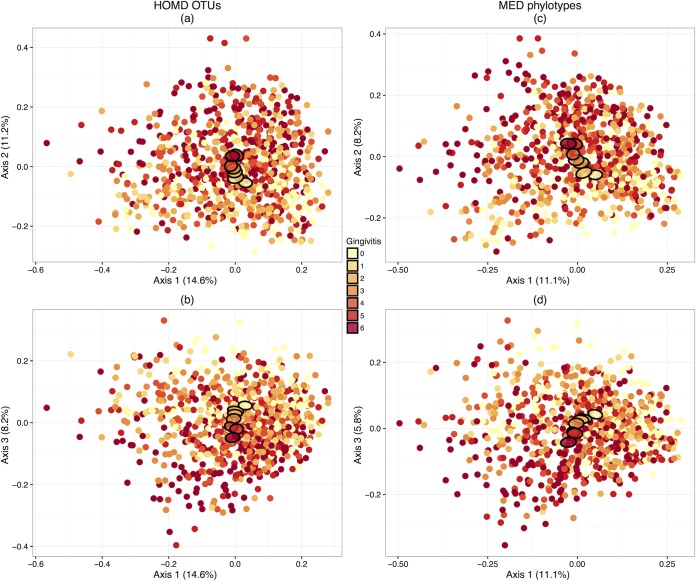
The PCoA ordination of supragingival plaque samples shows an approximate trend with gingivitis severity that is robust to analysis methods. PCoA ordinations based on Bray-Curtis dissimilarities between samples for 626 HOMD OTUs (a, b) and 502 MED phylotypes (c, d). Filled ellipses show mean values for each gingivitis severity, ranging from 0 (yellow) to 6 (dark red). In both cases, an approximate trend is visible, despite the noisiness of the data set. Before plotting, samples were rarefied to 5,000 reads to minimize the impact of sequencing depth.

Initial exploratory analysis with principal-coordinate analysis (PCoA) ordinations showed that, although there was large variability in community composition across supragingival plaque samples, there was also a clear trend related to gingivitis severity that was robust to the analysis method used (HOMD OTUs or MED phylotypes) (Fig. 1). Stratifying by periodontitis in the same way did not indicate visually clear differences.

**FIG 2:**
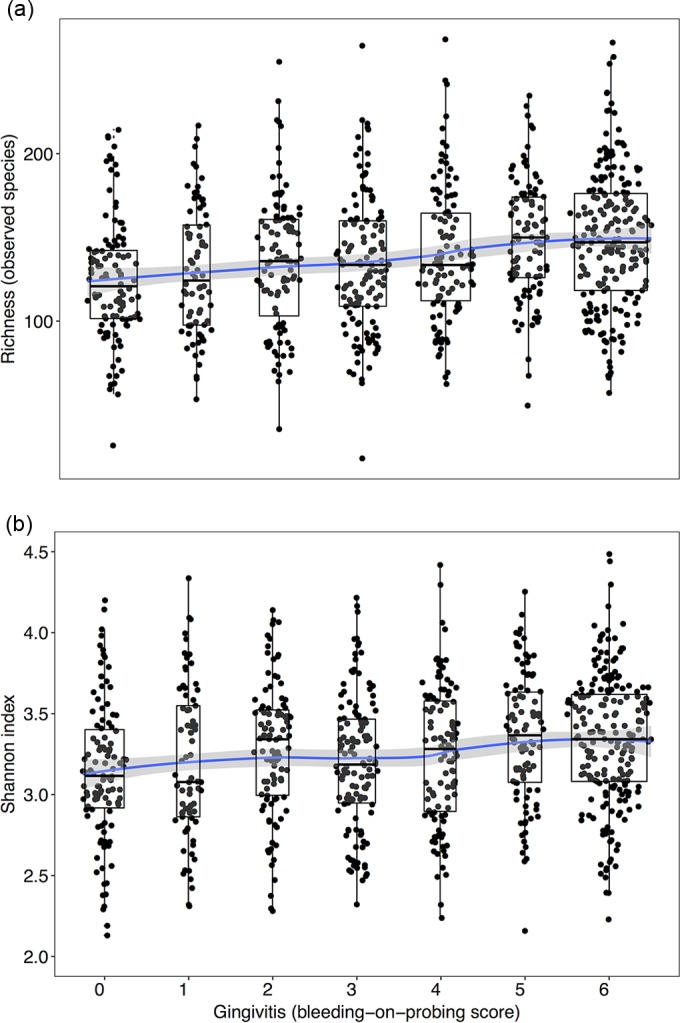
Microbial community richness and Shannon index increase with gingivitis severity. Both richness (number of observed species) (a) and Shannon index (measure of diversity) (b) of supragingival plaque increase with gingivitis severity. Estimates for each sample were calculated by sampling with replacement at a rarefaction depth of 5,000 sequences per sample and averaging over 100 iterations. The fitted line shows a local polynomial regression fit calculated using loess in R, with the gray region indicating the 95% CI. A total of 138/965 samples were excluded due to having fewer than 5,000 sequences. Changing the rarefaction depth did not affect the conclusion that gingivitis severity was associated with an increase in both species richness and Shannon index.

A quantitative analysis of diversity reflected this trend. Gingivitis was associated with higher microbial community richness (Fig. 2a) and Shannon indexes (Fig. 2b). Microbial communities did not markedly differ between healthy women and those with low levels of gingivitis. Both gingivitis and periodontitis were associated with higher supragingival plaque richness in a linear regression, controlling for demographic variables (see Table S3a in the supplemental material). In the final model predicting Shannon index, periodontitis was not retained but gingivitis was (see Table S3b in the supplemental material). Reversing the analysis, richness was retained in the final model for predicting gingivitis but not periodontitis (see Table S2 in the supplemental material).

### Differences in bacterial abundances with gingivitis.

Differential abundance analyses with DESeq2 (26, 27) found 118 OTUs that were significantly (false-discovery rate [lsqb*q*] < 0.05) associated with a greater severity of gingivitis (see Data Set S1 in the supplemental material), making up 16.6% of the data set in terms of reads. Conversely, 47 OTUs were associated with lower severity (18.7% of the data set), implying that gingivitis is not only related to bacterial load but also to the nature of the microbial community.

**FIG 3:**
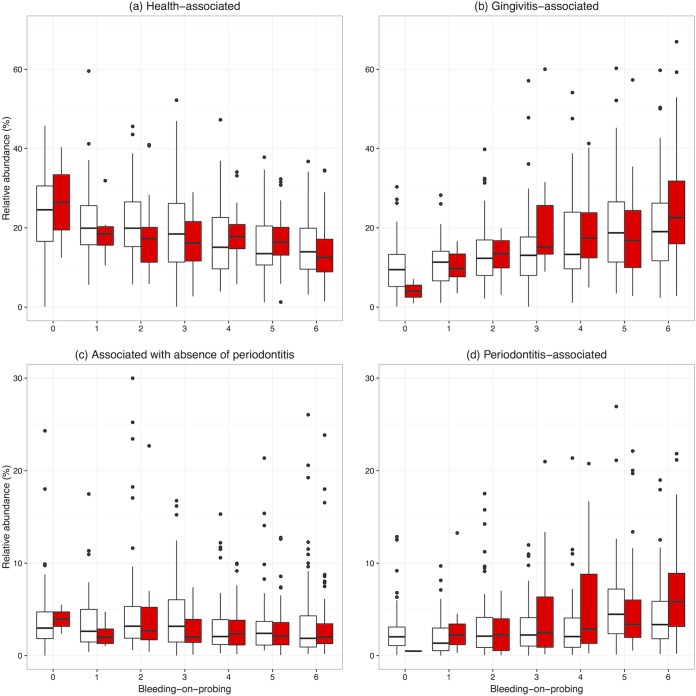
Summed percentage abundances of OTUs associated with decreased gingivitis (a), increased gingivitis (b), absence of periodontitis (c), and presence of periodontitis (d) for each periodontal disease category. For plotting purposes, samples were rarefied to 10,000 reads per sample, resulting in the removal of 269/962 samples; this rarefaction was not used in the selection of the OTUs, which was performed using DESeq2 on the whole data set. One outlier and two outliers in panels c and d, respectively, are not shown due to trimming the *y* axis at a relative abundance of 30%.

Figure 3a and b show the cumulative abundances of health- and gingivitis-associated OTUs, respectively, showing the progressive nature of changes with the degree of bleeding. Most of the pairwise comparisons of summed abundances of health- and gingivitis-associated OTUs were not significantly different between women with and without periodontitis (Kruskal-Wallis test, *P* > 0.05). However, for women with periodontitis, the severity of gingivitis was important, as there were microbial differences between women with and without periodontitis for both moderate gingivitis (BoP of 3; *P* = 0.014) and severe gingivitis (BoP of 6; *P* = 0.011). The most significantly gingivitis-associated OTU was Peptostreptococcus stomatis, which was present in over 75% of samples across severity categories and was an average of 1.45-fold more abundant (95% CI of 1.37 to 1.54) with a unit increase in BoP.

### Differences in bacterial abundances with periodontitis.

While gingivitis had a stronger association with supragingival microbiota, there were also differences in microbial community compositions with periodontitis (Fig. 3c and d). Seventy-one OTUs were significantly (*q* < 0.05) more abundant in women with periodontitis (see Data Set S1 in the supplemental material), making up 4.4% of the data set in terms of reads. Thirteen OTUs were significantly more abundant in the absence of periodontitis, making up 3.6% of the data set by reads. These health-associated OTUs were Lautropia mirabilis, Rothia aeria, Streptococcus pyogenes, Streptococcus mutans, and seven members of Actinomyces.

At the genus level for periodontitis-associated OTUs, Prevotella (14 OTUs) and Treponema (10 OTUs) were the most represented. Only one member of the pathogenic red complex (8) was significantly associated with periodontitis, Treponema denticola. The other two members (Porphyromonas gingivalis and Tannerella forsythia) were additionally not identified as MED phylotypes in the data set, which is possibly due to primer mismatch (see the discussion in the supplemental material). Eubacterium nodatum, previously identified as clustering with the red complex in supragingival plaque (45), was significantly associated with periodontitis.

### Differences in bacterial abundances unique to periodontitis.

Forty out of 71 periodontitis-associated OTUs (56%) were not associated with gingivitis (see Table S4 in the supplemental material). These taxa were rare; their mean cumulative abundance was 2.2%, with only six OTUs having mean relative abundances of >0.1%. The most represented genera were Prevotella (9 OTUs), Treponema (5 OTUs), and Selenomonas (4 OTUs).

The presence or absence of periodontitis was not a significant determinant of the cumulative abundances of these OTUs for women with the same levels of gingivitis (Kruskal-Wallis test, *P* > 0.05), except for women with a BoP of 4 (*P* = 0.026).

### The cooccurrence network of periodontitis-associated taxa.

The above analysis considers each OTU as independent, but in reality, oral bacteria exist in complex polymicrobial biofilms where interactions are extremely important (28). Cooccurrence analysis can allow for the identification of important members of microbial communities (46). We therefore analyzed the cooccurrence networks of periodontitis-associated bacteria across all periodontal severities.

A preliminary network analysis of periodontitis-associated OTUs across periodontal severities indicated that the network was more connected in women with periodontitis across gingivitis severities (see Fig. S1 in the supplemental material). However, we sought to confirm this cooccurrence pattern with a higher resolution analysis. We therefore selected all MED phylotypes that had >98.5% similarity to a periodontitis-associated OTU (see Materials and Methods). Eighty-one MED phylotypes had representative sequences with >98.5% similarity to a periodontitis-associated OTU (see Data Set S2 in the supplemental material).

**FIG 4:**
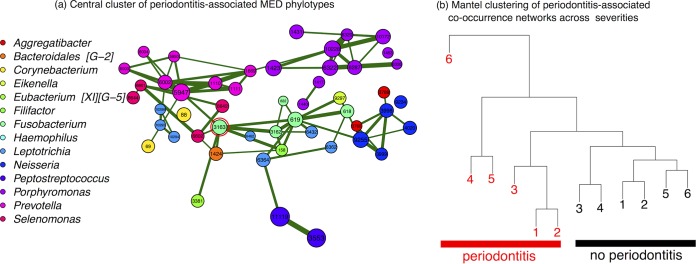
The cooccurrence network of periodontitis-associated bacteria shows a distinct community structure with the presence of periodontitis across gingivitis severities. (a) The strongly connected central cooccurrence network of periodontitis-associated bacteria across supragingival plaque samples from *n =* 110 women with severe gingivitis (BoP = 6) and periodontitis. Shown here are significant strong pairwise Spearman correlation coefficients (*P* < 0.01; ρ > 0.405), calculated with SparCC between MED phylotypes with >98.5% similarity to periodontitis-associated HOMD OTUs (see Materials and Methods). Node color indicates taxonomic genus, size is proportional to log-transformed mean relative abundance, and edge weight indicates the strength of the correlation. The red circle indicates the node with the highest betweenness centrality, classified taxonomically as Fusobacterium nucleatum subsp. vincentii. Node layout was determined using the Fruchterman-Reingold algorithm in qgraph v1.3.1. Twenty-two nodes without any strong correlations connecting them to the rest of the network (i.e., no edges with a ρ of >0.405) were removed during figure preparation. (b) Clustering using hclust in R of the correlation matrices calculated in this way for all severities of periodontal disease. The periodontitis-associated cooccurrence network is more similar between women with periodontitis, regardless of gingivitis severity. Correlation matrices were not adjusted for significance due to the different numbers of women between groups.

The strongly connected cooccurrence network in women with severe gingivitis (BOP of 6) and periodontitis showed several genus-level clusters, including Selenomonas, Peptostreptococcus, and Prevotella (Fig. 4a). Notably, these clusters were connected by a small group of central bacteria, including Filifactor alocis (phylotype 158) and several members of Fusobacterium nucleatum with phylotypes classified taxonomically as subspecies vincentii (phylotypes 3163 and 622) and polymorphum (phylotypes 618 and 619), suggesting their roles in the coaggregation of periodontal biofilms. Ranking phylotypes in the strongly connected network according to their betweenness centrality, which measures the potential for influence on information transfer in a network (47), the most connected phylotype was F. nucleatum subsp. vincentii (phylotype 3163) (see Table S5 in the supplemental material). T. denticola was not present in this network, but when MED analysis was repeated with the minimum substantive abundance parameter reduced by a factor of 10 to 0.01%, we found that it was placed in the network in a central position.

To confirm that this altered community structure was a distinguishing feature of supragingival plaque between women with and without periodontitis, we clustered the correlation matrices based on Mantel distances for each category of periodontal disease (Fig. 4b). Networks clustered by the periodontitis status of the women in the group, which confirmed that the altered community structure with periodontitis was detectable even in women with low levels of gingivitis. Within the periodontitis groupings, matrices clustered by gingivitis severity.

## DISCUSSION

In this study, we investigated changes in the supragingival microbiome associated with periodontal disease severity in a large cross-sectional cohort in Malawi. Our main finding was that even though the composition of supragingival plaque is primarily associated with gingivitis, as quantified by bleeding on probing, rather than the presence or absence of periodontitis, the presence of periodontitis has detectable associations with supragingival microbiota that are unrelated to gingivitis. In particular, the differences in cooccurrence patterns of taxa between women with and without periodontitis support a more complex etiology of disease than a simple progression from health through gingivitis to periodontitis.

Gingivitis and periodontitis were associated with higher microbial community richness and Shannon indexes, and this association remained after adjustment for demographic factors, including age, body mass index (BMI), and socioeconomic status. This finding is consistent with previous research (48, 49), with higher diversity meaning that, in periodontal disease, the oral microbiota is added rather than existing taxa undergoing replacement. This may correspond to primary ecological succession in a new environmental niche, as suggested by Abusleme et al. (50).

We found that many taxa were associated with gingivitis and periodontitis. The abundance of the majority of these taxa increased with gingivitis severity, and this pattern was not influenced by the presence of periodontitis. Furthermore, some women without gingivitis had similar summed percentage abundances of disease-associated taxa compared to women with severe gingivitis. It would appear that relative bacterial abundances alone are insufficient to explain the presence of disease, which is consistent with a requirement for other factors, such as the host inflammatory response, to cause disease.

Periodontitis-associated OTUs were also identified, including known periodontal pathogens like F. alocis, T. denticola, F. nucleatum, and P. stomatis, which is consistent with findings from other populations (28). OTUs, including members of Prevotella, Treponema, and Selenomonas, were not significantly associated with gingivitis severity, supporting the idea that periodontitis is not just an advanced phase of gingivitis and involves additional bacteria. However, cumulative abundances of periodontitis-associated OTUs did not differ significantly between women with and without periodontitis who had the same levels of gingivitis, which suggests that abundances do not fully explain the disease.

What we did observe were different cooccurrence patterns across disease categories for periodontitis-associated bacteria, which indicated the presence of a consistent community structure in women with periodontitis across all gingivitis severities. Central nodes in this periodontitis-associated network included F. alocis and several subspecies of F. nucleatum, which acted as hubs connecting different clusters. Network analysis using betweenness centrality ranked F. nucleatum subsp. vincentii (phylotype 3163) as the most central phylotype in the strongly connected cooccurrence network in women with severe gingivitis and periodontitis. These findings are consistent with the proposed roles of “bridging bacteria” that contribute to the coaggregation of periodontal biofilms (51). F. nucleatum has been shown experimentally to facilitate the survival of obligate anaerobes in aerated environments (52) and has been identified as one of the important precursors to attachment by later colonizers in periodontal disease (51). F. alocis has also been experimentally linked to the coaggregation of periodontal biofilms (53, 54) and correlates with the greater inflammation in periodontitis (24). Chen et al. also identified a similar F. alocis-centered cooccurrence group of taxa that was enriched in multiple oral habitats during periodontitis compared with those in healthy controls (49).

### Limitations.

The main strength of this study is that we were able to include women with different severities and combinations of periodontal disease, allowing us to distinguish signals from gingivitis and periodontitis. However, our observations about periodontitis only apply to supragingival plaque, as we did not sample from subgingival plaque due to the difficulty of collecting such a large number of samples from a cohort in a resource-limited setting. However, previous work has shown that sampling supragingival plaque still allows for the detection of bacteria associated with periodontitis while being minimally invasive and simple to perform (55). Similarly, we were able to observe changes in the abundances of rare taxa that were known to be associated with the subgingival plaque of periodontitis. For example, Fretibacterium fastidiosum (HOMD identification 360BH017), which accounted for a mean of just 0.009% of reads, was still significantly more abundant (2.5-fold) in women with periodontitis, which is consistent with the recent finding of a higher abundance in subgingival plaque when periodontitis was compared to gingivitis (23).

Another limitation was that samples were collected from across the mouth instead of localizing sampling to sites of specific interest. The distribution of bacterial species across the mouth is known to be heterogeneous, with supragingival plaque at sites adjacent to deepened periodontal pockets showing significantly higher counts of periodontitis-associated species (45). Due to the size of our cohort, we used a single swab, which was probably responsible for the large amount of variability in our data set when visualized in ordinations (Fig. 1), and effectively pooled all supragingival sites. This precluded an investigation of heterogeneity between sites, but detectable associations with both gingivitis and periodontitis were still present even with this approach.

We treated gingivitis as a continuous variable but periodontitis as binary. In reality, periodontitis is a complex disease with a problematic classification (15), and it is likely that our simple treatment of periodontitis obscures this complexity. This may cause bacterial cooccurrence patterns in women with periodontitis to appear stronger, as women with more severe disease may have greater abundances of associated bacterial species.

Our study is the largest to be conducted so far in a sub-Saharan population, and our results appear to be consistent, for the most part, with previous work on bacterial associations with periodontal disease (16, 28, 45, 49, 56). However, it should be pointed out that our population was additionally notable in two respects. First, all participants were women who had recently given birth. Pregnancy, particularly in its early to middle stages, is known to be linked to periodontal disease and potential changes in the oral microbiome (57), with an increased susceptibility to gingivitis (58), although subgingival levels of known periodontal pathogens may remain unchanged (59). Qualitative differences between periodontal pathogens found during pregnancy and postpartum have also been observed (60). It is not clear for how long after pregnancy the oral microbiome remains altered, but evidence that significant changes are mainly detectable in early pregnancy (57) and the consistency of our results with other studies suggest that effects remaining after 6 weeks postpartum are small. Second, all women in the study were intermittently given sulfadoxine-pyrimethamine (SP) at enrollment and between the 28th and 34th gestational week for malaria prevention. Since systemic antibiotics can be given as a treatment for aggressive periodontitis (61), patients who have received antibiotic treatment in the previous 6 months are often excluded from studies of periodontitis. However, the salivary microbiome has been shown to be robust to disturbance by a week-long course of antibiotics (62). Given that SP treatment was intermittent, involved antibiotics not targeted at periodontal bacteria, and took place around 2 months before the oral sampling, we believe that it is unlikely to have played an important role but have no direct evidence to support this claim.

### Conclusion.

This study represents the largest to date investigating associations between supragingival plaque composition and various severities of periodontal disease in a low-income sub-Saharan population with limited oral hygiene. We have identified distinct signals associated with gingivitis and periodontitis in supragingival plaque, with a dominant contribution from gingivitis. Future proposals for a diagnostic test for periodontitis based on supragingival plaque sampling, which may be useful in low-resource settings, will need to take this into account. Network analysis of observed cooccurrence patterns was consistent with the role of bridging bacteria like F. nucleatum and F. alocis in the coaggregation of periodontal biofilms prior to penetrance into subgingival regions. Although some periodontitis-associated bacteria were also associated with gingivitis, the major change with periodontitis is in the network of cooccurrences. Viewed this way, gingivitis sets the stage for periodontitis to develop by providing an environment where periodontitis-associated taxa can increase in abundance and coaggregate into pathogenic biofilms that may then penetrate to subgingival regions. More quantitative modeling of associations between oral bacteria and various clinical features of disease will be necessary to understand these complex relationships and explore the microbial ecology of periodontitis.

## Supplementary Material

Supplemental material

## References

[1] Petersen PE, Bourgeois D, Ogawa H, Estupinan-Day S, Ndiaye C 2005 The global burden of oral diseases and risks to oral health. Bull World Health Organ 83:661–669. 16211157 PMC2626328

[2] Li X, Kolltveit KM, Tronstad L, Olsen I 2000 Systemic diseases caused by oral infection. Clin Microbiol Rev 13:547–558. doi:10.1128/CMR.13.4.547-558.2000. 11023956 PMC88948

[3] Yu Y-H, Chasman DI, Buring JE, Rose L, Ridker PM 2015 Cardiovascular risks associated with incident and prevalent periodontal disease. J Clin Periodontol 42:21–28. doi:10.1111/jcpe.12335. 25385537 PMC4300240

[4] Ide M, Papapanou PN 2013 Epidemiology of association between maternal periodontal disease and adverse pregnancy outcomes—systematic review. J Periodontol 84:S181–S194. doi:10.1902/jop.2013.134009. 23631578

[5] Hajishengallis G 2015 Periodontitis: from microbial immune subversion to systemic inflammation. Nat Rev Immunol 15:30–44. doi:10.1038/nri3785. 25534621 PMC4276050

[6] Baelum V, Scheutz F 2002 Periodontal diseases in Africa. Periodontol 2000 29:79–103. doi:10.1034/j.1600-0757.2002.290105.x. 12102704

[7] Van Dyke TE 2008 The management of inflammation in periodontal disease. J Periodontol 79:1601–1608. doi:10.1902/jop.2008.080173. 18673016 PMC2563957

[8] Socransky SS, Haffajee AD, Cugini MA, Smith C, Kent RL 1998 Microbial complexes in subgingival plaque. J Clin Periodontol 25:134–144. doi:10.1111/j.1600-051X.1998.tb02419.x. 9495612

[9] Jiao Y, Hasegawa M, Inohara N 2014 The role of oral pathobionts in dysbiosis during periodontitis development. J Dent Res 93:539–546. doi:10.1177/0022034514528212. 24646638 PMC4023464

[10] Wade WG 2013 The oral microbiome in health and disease. Pharmacol Res 69:137–143. doi:10.1016/j.phrs.2012.11.006. 23201354

[11] Schätzle M, Löe H, Bürgin W, Anerud A, Boysen H, Lang NP 2003 Clinical course of chronic periodontitis. I. Role of gingivitis. J Clin Periodontol 30:887–901. doi:10.1034/j.1600-051X.2003.00414.x. 14710769

[12] Batchelor P 2014 Is periodontal disease a public health problem? Br Dent J 217:405–409. doi:10.1038/sj.bdj.2014.912. 25342346

[13] Jeffcoat MK, Reddy MS 1991 Progression of probing attachment loss in adult periodontitis. J Periodontol 62:185–189. doi:10.1902/jop.1991.62.3.185. 2027069

[14] Haffajee AD, Socransky SS 1986 Attachment level changes in destructive periodontal diseases. J Clin Periodontol 13:461–475. doi:10.1111/j.1600-051X.1986.tb01491.x. 3522651

[15] Mdala I, Olsen I, Haffajee AD, Socransky SS, Thoresen M, de Blasio BF 2014 Comparing clinical attachment level and pocket depth for predicting periodontal disease progression in healthy sites of patients with chronic periodontitis using multi-state Markov models. J Clin Periodontol 41:837–845. doi:10.1111/jcpe.12278. 24888705 PMC4139458

[16] Ximénez-Fyvie LA, Haffajee AD, Socransky SS 2000 Microbial composition of supra- and subgingival plaque in subjects with adult periodontitis. J Clin Periodontol 27:722–732. doi:10.1034/j.1600-051x.2000.027010722.x. 11034118

[17] Ximénez-Fyvie LA, Haffajee AD, Socransky SS 2000 Comparison of the microbiota of supra- and subgingival plaque in health and periodontitis. J Clin Periodontol 27:648–657. doi:10.1034/j.1600-051x.2000.027009648.x. 10983598

[18] Haffajee AD, Teles RP, Patel MR, Song X, Veiga N, Socransky SS 2009 Factors affecting human supragingival biofilm composition. I. Plaque mass. J Periodont Res 44:511–519. doi:10.1111/j.1600-0765.2008.01154.x. 18973540 PMC2710401

[19] Griffen AL, Beall CJ, Firestone ND, Gross EL, Difranco JM, Hardman JH, Vriesendorp B, Faust RA, Janies DA, Leys EJ 2011 CORE: a phylogenetically-curated 16S rDNA database of the core oral microbiome. PLoS One 6:e19051. doi:10.1371/journal.pone.0019051. 21544197 PMC3081323

[20] Chen T, Yu W-H, Izard J, Baranova OV, Lakshmanan A, Dewhirst FE 2010 The Human Oral Microbiome Database: a web accessible resource for investigating oral microbe taxonomic and genomic information. Database (Oxford) 2010:baq013. 20624719 10.1093/database/baq013 PMC2911848

[21] Stearns JC, Lynch MDJ, Senadheera DB, Tenenbaum HC, Goldberg MB, Cvitkovitch DG, Croitoru K, Moreno-Hagelsieb G, Neufeld JD 2011 Bacterial biogeography of the human digestive tract. Sci Rep 1:170. 22355685 10.1038/srep00170 PMC3240969

[22] Huttenhower C, Gevers D, Knight R, Abubucker S, Badger JH, Chinwalla AT, Creasy HH, Earl AM, FitzGerald MG, Fulton RS, Giglio MG, Hallsworth-Pepin K, Lobos EA, Madupu R, Magrini V, Martin JC, Mitreva M, Muzny DM, Sodergren EJ, Versalovic J, Wollam AM, Worley KC, Wortman JR, Young SK, Zeng Q, Aagaard KM, Abolude OO, Allen-Vercoe E, Alm EJ, Alvarado L, Andersen GL, Anderson S, Appelbaum E, Arachchi HM, Armitage G, Arze CA, Ayvaz T, Baker CC, Begg L, Belachew T, Bhonagiri V, Bihan M, Blaser MJ, Bloom T, Bonazzi V, Brooks J, Buck GA, Buhay CJ, Busam DA, Campbell JL, 2012 Structure, function and diversity of the healthy human microbiome. Nature 486:207–214. doi:10.1038/nature11234. 22699609 PMC3564958

[23] Park O-J, Yi H, Jeon JH, Kang S-S, Koo K-T, Kum K-Y, Chun J, Yun C-H, Han SH 2015 Pyrosequencing analysis of subgingival microbiota in distinct periodontal conditions. J Dent Res 94:921–927. doi:10.1177/0022034515583531. 25904141

[24] Camelo-Castillo A, Novoa L, Balsa-Castro C, Blanco J, Mira A, Tomás I 2015 Relationship between periodontitis-associated subgingival microbiota and clinical inflammation by 16S pyrosequencing. J Clin Periodontol 42:1074–1082. doi:10.1111/jcpe.12470. 26461079

[25] Ashorn P, Alho L, Ashorn U, Cheung YB, Dewey KG, Harjunmaa U, Lartey A, Nkhoma M, Phiri N, Phuka J, Vosti SA, Zeilani M, Maleta K 2015 The impact of lipid-based nutrient supplement provision to pregnant women on newborn size in rural Malawi: a randomized controlled trial. Am J Clin Nutr 101:387–397. doi:10.3945/ajcn.114.088617. 25646337

[26] Love MI, Huber W, Anders S 2014 Moderated estimation of fold change and dispersion for RNA-seq data with DESeq2. Genome Biol 15:550. doi:10.1186/s13059-014-0550-8. 25516281 PMC4302049

[27] McMurdie PJ, Holmes S 2014 Waste not, want not: why rarefying microbiome data is inadmissible. PLoS Comput Biol 10:e1003531. doi:10.1371/journal.pcbi.1003531. 24699258 PMC3974642

[28] Teles R, Teles F, Frias-Lopez J, Paster B, Haffajee A 2013 Lessons learned and unlearned in periodontal microbiology. Periodontol 2000 62:95–162. doi:10.1111/prd.12010. 23574465 PMC3912758

[29] Harjunmaa U, Järnstedt J, Alho L, Dewey KG, Cheung YB, Deitchler M, Ashorn U, Maleta K, Klein NJ, Ashorn P 2015 Association between maternal dental periapical infections and pregnancy outcomes: results from a cross-sectional study in Malawi. Trop Med Int Health 20:1549–1558. doi:10.1111/tmi.12579. 26224026

[30] Bonder MJ, Abeln S, Zaura E, Brandt BW 2012 Comparing clustering and pre-processing in taxonomy analysis. Bioinformatics 28:2891–2897. doi:10.1093/bioinformatics/bts552. 22962346

[31] Doyle RM, Alber DG, Jones HE, Harris K, Fitzgerald F, Peebles D, Klein N 2014 Term and preterm labour are associated with distinct microbial community structures in placental membranes which are independent of mode of delivery. Placenta 35:1099–1101. doi:10.1016/j.placenta.2014.10.007. 25458966

[32] Caporaso JG, Lauber CL, Walters WA, Berg-Lyons D, Huntley J, Fierer N, Owens SM, Betley J, Fraser L, Bauer M, Gormley N, Gilbert JA, Smith G, Knight R 2012 Ultra-high-throughput microbial community analysis on the Illumina HiSeq and MiSeq platforms. ISME J 6:1621–1624. doi:10.1038/ismej.2012.8. 22402401 PMC3400413

[33] Caporaso JG, Kuczynski J, Stombaugh J, Bittinger K, Bushman FD, Costello EK, Fierer N, Peña AG, Goodrich JK, Gordon JI, Huttley GA, Kelley ST, Knights D, Koenig JE, Ley RE, Lozupone CA, McDonald D, Muegge BD, Pirrung M, Reeder J, Sevinsky JR, Turnbaugh PJ, Walters WA, Widmann J, Yatsunenko T, Zaneveld J, Knight R 2010 QIIME allows analysis of high-throughput community sequencing data. Nat Methods 7:335–336. doi:10.1038/nmeth.f.303. 20383131 PMC3156573

[34] Edgar RC 2010 Search and clustering orders of magnitude faster than BLAST. Bioinformatics 26:2460–2461. doi:10.1093/bioinformatics/btq461. 20709691

[35] Westcott SL, Schloss PD 2015 *De novo* clustering methods outperform reference-based methods for assigning 16S rRNA gene sequences to operational taxonomic units. PeerJ 3:e1487. doi:10.7717/peerj.1487. 26664811 PMC4675110

[36] Eren AM, Morrison HG, Lescault PJ, Reveillaud J, Vineis JH, Sogin ML 2015 Minimum entropy decomposition: unsupervised oligotyping for sensitive partitioning of high-throughput marker gene sequences. ISME J 9:968–979. 25325381 10.1038/ismej.2014.195 PMC4817710

[37] Eren AM, Maignien L, Sul WJ, Murphy LG, Grim SL, Morrison HG, Sogin ML 2013 Oligotyping: differentiating between closely related microbial taxa using 16S rRNA gene data. Methods Ecol Evol 4:1111–1119. doi:10.1111/2041-210X.12114. PMC3864673 24358444

[38] Rognes T 2016 Vsearch. University of Oslo, Oslo, Norway.

[39] Huse SM, Dethlefsen L, Huber JA, Welch DM, Relman DA, Sogin ML 2008 Exploring microbial diversity and taxonomy using SSU rRNA hypervariable tag sequencing. PLoS Genet 4:e1000255. doi:10.1371/journal.pgen.1000255. 19023400 PMC2577301

[40] Akaike H 1974 A new look at the statistical model identification. IEEE Trans Automat Contr 19:716–723. doi:10.1109/TAC.1974.1100705.

[41] Benjamini Y, Hochberg Y 1995 Controlling the false discovery rate: a practical and powerful approach to multiple testing. J R Stat Soc Ser B 57:289–300.

[42] Friedman J, Alm EJ 2012 Inferring correlation networks from genomic survey data. PLoS Comput Biol 8:e1002687. doi:10.1371/journal.pcbi.1002687. 23028285 PMC3447976

[43] Epskamp S, Cramer AOJ, Waldorp LJ, Schmittmann VD, Borsboom D 2012 qgraph: network visualizations of relationships in psychometric data. J Stat Softw 48:1–18.

[44] Fruchterman TMJ, Reingold EM 1991 Graph drawing by force-directed placement. Softw Pract Exp 21:1129–1164. doi:10.1002/spe.4380211102.

[45] Haffajee AD, Socransky SS, Patel MR, Song X 2008 Microbial complexes in supragingival plaque. Oral Microbiol Immunol 23:196–205. doi:10.1111/j.1399-302X.2007.00411.x. 18402605

[46] Faust K, Lahti L, Gonze D, de Vos WM, Raes J 2015 Metagenomics meets time series analysis: unraveling microbial community dynamics. Curr Opin Microbiol 25:56–66. doi:10.1016/j.mib.2015.04.004. 26005845

[47] Freeman LC 1977 A set of measures of centrality based on betweenness. Sociometry 40:35–41. doi:10.2307/3033543.

[48] Kistler JO, Booth V, Bradshaw DJ, Wade WG 2013 Bacterial community development in experimental gingivitis. PLoS One 8:e71227. doi:10.1371/journal.pone.0071227. 23967169 PMC3743832

[49] Chen H, Liu Y, Zhang M, Wang G, Qi Z, Bridgewater L, Zhao L, Tang Z, Pang X 2015 A Filifactor alocis-centered co-occurrence group associates with periodontitis across different oral habitats. Sci Rep 5:9053. doi:10.1038/srep09053. 25761675 PMC4356962

[50] Abusleme L, Dupuy AK, Dutzan N, Silva N, Burleson JA, Strausbaugh LD, Gamonal J, Diaz PI 2013 The subgingival microbiome in health and periodontitis and its relationship with community biomass and inflammation. ISME J 7:1016–1025. doi:10.1038/ismej.2012.174. 23303375 PMC3635234

[51] Aruni AW, Dou Y, Mishra A, Fletcher HM 2015 The biofilm community—rebels with a cause. Curr Oral Heal Rep 2:48–56. doi:10.1007/s40496-014-0044-5. PMC4478205 26120510

[52] Bradshaw DJ, Marsh PD, Watson GK, Allison C 1998 Role of Fusobacterium nucleatum and coaggregation in anaerobe survival in planktonic and biofilm oral microbial communities during aeration. Infect Immun 66:4729–4732. 9746571 10.1128/iai.66.10.4729-4732.1998 PMC108582

[53] Schlafer S, Riep B, Griffen AL, Petrich A, Hübner J, Berning M, Friedmann A, Göbel UB, Moter A 2010 Filifactor alocis–involvement in periodontal biofilms. BMC Microbiol 10:66. doi:10.1186/1471-2180-10-66. 20193074 PMC2846919

[54] Fine DH, Markowitz K, Fairlie K, Tischio-Bereski D, Ferrendiz J, Furgang D, Paster BJ, Dewhirst FE 2013 A consortium of Aggregatibacter actinomycetemcomitans, Streptococcus parasanguinis, and Filifactor alocis is present in sites prior to bone loss in a longitudinal study of localized aggressive periodontitis. J Clin Microbiol 51:2850–2861. doi:10.1128/JCM.00729-13. 23784124 PMC3754677

[55] Galimanas V, Hall MW, Singh N, Lynch MDJ, Goldberg M, Tenenbaum H, Cvitkovitch DG, Neufeld JD, Senadheera DB 2014 Bacterial community composition of chronic periodontitis and novel oral sampling sites for detecting disease indicators. Microbiome 2:32. doi:10.1186/2049-2618-2-32. 25225610 PMC4164120

[56] Haffajee AD, Socransky SS 1994 Microbial etiological agents of destructive periodontal diseases. Periodontol 2000 5:78–111. doi:10.1111/j.1600-0757.1994.tb00020.x. 9673164

[57] Fujiwara N, Tsuruda K, Iwamoto Y, Kato F, Odaki T, Yamane N, Hori Y, Harashima Y, Sakoda A, Tagaya A, Komatsuzawa H, Sugai M, Noguchi M 8 9 2015 Significant increase of oral bacteria in the early pregnancy period in Japanese women. J Investig Clin Dent doi:10.1111/jicd.12189. 26345599

[58] Gürsoy M, Könönen E, Gürsoy UK, Tervahartiala T, Pajukanta R, Sorsa T 2010 Periodontal status and neutrophilic enzyme levels in gingival crevicular fluid during pregnancy and postpartum. J Periodontol 81:1790–1796. doi:10.1902/jop.2010.100147. 20831370

[59] Adriaens LM, Alessandri R, Spörri S, Lang NP, Persson GR 2009 Does pregnancy have an impact on the subgingival microbiota? J Periodontol 80:72–81. doi:10.1902/jop.2009.080012. 19228092

[60] Carrillo-de-Albornoz A, Figuero E, Herrera D, Bascones-Martínez A 2010 Gingival changes during pregnancy: II. Influence of hormonal variations on the subgingival biofilm. J Clin Periodontol 37:230–240. 20088983 10.1111/j.1600-051X.2009.01514.x

[61] Rabelo CC, Feres M, Gonçalves C, Figueiredo LC, Faveri M, Tu Y-K, Chambrone L 2015 Systemic antibiotics in the treatment of aggressive periodontitis. A systematic review and a Bayesian network meta-analysis. J Clin Periodontol 42:647–657. 26087839 10.1111/jcpe.12427

[62] Zaura E, Brandt BW, Teixeira de Mattos MJ, Buijs MJ, Caspers MPM, Rashid M-U, Weintraub A, Nord CE, Savell A, Hu Y, Coates AR, Hubank M, Spratt DA, Wilson M, Keijser BJF, Crielaard W 2015 Same exposure but two radically different responses to antibiotics: resilience of the salivary microbiome versus long-term microbial shifts in feces. mBio 6:e01693-15. doi:10.1128/mBio.01693-15. PMC4659469 26556275

