# Supplementary material for: Distinguishing the Signals of Gingivitis and Periodontitis in Supragingival Plaque: a Cross-Sectional Cohort Study in Malawi
Source: Appl Environ Microbiol. 2016 Sep 16;82(19):6057–67. doi: 10.1128/AEM.01756-16 (PMC5038043; doi:10.1128/AEM.01756-16)
Supplement: Supplemental material [file supp_82_19_6057__index.html]

Supplemental material 

# Distinguishing the Signals of Gingivitis and Periodontitis in Supragingival Plaque: a Cross-Sectional Cohort Study in Malawi

## Supplemental material

- Supplemental file 1 -

  Supplemental text and discussion, as well as cooccurrence networks of periodontitis-associated taxa, which are more connected in women with periodontitis across gingivitis severities (Fig. S1).

  PDF, 470K
- Supplemental file 2 -

  Coefficients for regression models to predict gingivitis and periodontitis (Table S1).

  XLSX, 49K
- Supplemental file 3 -

  Regression models to predict gingivitis and periodontitis, including microbial community richness (Table S2).

  XLSX, 55K
- Supplemental file 4 -

  Linear regression models predicting richness and Shannon index of supragingival microbial communities (Table S3).

  XLSX, 47K
- Supplemental file 5 -

  OTUs significantly associated with periodontitis but not gingivitis (Table S4).

  XLSX, 56K
- Supplemental file 6 -

  Ranking of phylotypes in strongly connected cooccurrence network in women with severe gingivitis and periodontitis by their normalized betweenness centrality score (Table S5).

  XLSX, 53K
- Supplemental file 7 -

  DESeq results for gingivitis and periodontitis (Data Set S1).

  XLSX, 215K
- Supplemental file 8 -

  MED phylotypes (Data Set S2).

  XLSX, 118K
- Supplemental file 9 -

  HOMD taxa with mismatches with the 785F/1175R primer set used (Data Set S3).

  XLSX, 57K
