# Supplementary material for: Distinguishing the Signals of Gingivitis and Periodontitis in Supragingival Plaque: a Cross-Sectional Cohort Study in Malawi
Source: Appl Environ Microbiol. 2016 Sep 16;82(19):6057–67. doi: 10.1128/AEM.01756-16 (PMC5038043; doi:10.1128/AEM.01756-16)

Distinguishing the Signals of Gingivitis and Periodontitis in Supragingival Plaque: A Cross-Sectional Cohort Study in Malawi

Supplemental Material

Demographic characteristics predictive of periodontal disease

We fitted a linear regression model to predict gingivitis severity using selected demographic variables (Table 2) for 946/962 women without any missing data. After backwards stepwise elimination of variables using AIC as a criterion for model selection (1), the best model (see Table S1a) showed that gingivitis was more severe in older women (OR 1·06 per year; 95% CI 1·03-1·08) with lower BMI (0·96; 0·91-1·00), fewer years of education (0·91 per year; 0·87-0·95), a lower socio-economic status (0·71; 95% 0·61-0·84), and no malaria (0·73; 0·53-1·00). HIV was not included in the best model, in agreement with previous research that found no association with periodontal disease (2, 3).

We also applied the same procedure to predict (binary) periodontitis using a logistic regression model that included gingivitis severity. The best model (Table S2b) showed that periodontitis was more likely in women with more severe gingivitis (OR 1·68 per BoP; 95% CI 1·53-1·84) who were older (1·09 per year; 1·06-1·12), had fewer years of education (0·95 per year; 0·90-1·00) and a lower socio-economic status (0·85; 0·68-1·05) (Table S2b).

Addition of microbial community richness improves prediction of gingivitis but not periodontitis

To see if adding information on the diversity of supragingival plaque microbial communities improved the models, we added in the calculated richness to the full model to predict gingivitis and periodontitis for 811/962 women with >5,000 reads and no missing data, then again performed stepwise reduction according to AIC. Evenness of microbial communities was not included in the model due to high collinearity with richness (Spearman's rho=0·88).

Richness was retained in the final model for gingivitis (Table S2a) but not periodontitis (Table S2b). In this reduced set of data, HIV was retained in the final model for periodontitis (Table S2b), hence its inclusion as a potential confounder in subsequent differential abundance analysis.

Minimum Entropy Decomposition (MED) details

The table below gives information on the number of reads at each point in the filtering process prior to analysis with MED (4).

**Criteria**	**Reads remaining**
Maximum expected errors < 1	14,466,591

Minimum length 350	14,466,222
Maximum length 380	14,458,493
Samples <1,000 reads discarded	14,449,794

We than ran MED using the command:

```
decompose -M 1444 -V 3
```

The following table contains the output statistics:

Number of raw nodes (before the refinement)	502
Outliers removed due to -M	3,332,317
Outliers removed due to -V	1,012,339
Total number of outliers removed during the refinement	4,344,656
Number of samples found	946
Number of final nodes (after the refinement)	502
Number of sequences represented after quality filtering	10,105,138
Final number of outliers due to -M	3,332,317
Final number of outliers due to -V	1,012,339
Final total number of outliers	4,344,656

Primer mismatch and its effect on phylotype detection

We used the 785F/1175R primer pair to amplify the V5-V7 region of the 16S rRNA gene, following a standard protocol developed and used in previous studies (5, 6). These primer pairs have several degenerate positions indicated in **bold** (R = A/G, B = C/G/T, D = A/G/T):

785F: GGATTAGATACCC**BR**GTAGTC

1175R: ACGTC**R**TCCCC**D**CCTTCCTC

It is well established that different primer pairs can differentially amplify DNA from different taxa, biasing detection and subsequent analysis (7–9). Therefore, care should always be taken in interpreting marker gene data obtained using this approach: most importantly, absence of evidence is not the same as evidence of absence.

To identify phylotypes that we would expect to be less efficiently amplified by the primers, we searched all primers (2x3=6 possibilities for each primer) against the HOMD v13.2 database (10) with blastn v2.2.31 (11). This identified HOMD sequences that had mismatches with the primers. For the 785F and 1175R primers, there were 8 and 51 HOMD sequences respectively that did not have 100% similarity with one of the possible primers. These are given in Supplementary Dataset S3.

A priori, we would therefore expect phylotypes corresponding to these sequences to be absent (or detected at misleadlingly low levels) in our dataset, even if they were present in the original sample.

In particular, this list of phylotypes includes the well-established periodontal pathogens *Porphyromonas gingivalis* and *Tannerella forsythia* (12). Therefore, the fact that these pathogens are absent from our dataset is possibly due to the mismatch between the relevant regions of their 16S rRNA genes and the 1175R primer and should not be interpreted as proof that they are not associated with periodontal disease in Malawian women.

Co-occurrence network preparation

Co-occurrence network analysis using HOMD OTUs associated with periodontitis showed more connections in the network in women with periodontitis across gingivitis severities (Figure S1). However, we wanted to verify this result with MED analysis to ensure co-occurrence patterns were not due to the limited resolution of the OTU picking process.

Therefore, we selected all 81 MED phylotypes with >98.5% sequence similarity to periodontitis-associated HOMD OTUs. However, this included 19 members of *Streptococcus*, despite the fact that only *S. oligofermentans* (HOT 886) was associated with periodontitis, due to the high sequence similarity of this genus in the V5-V7 region. When plotted as a co-occurrence network, these phylotypes clearly clustered away from the periodontitis-associated phylotypes and had negative correlations with the rest of the network. We therefore removed them when preparing Figure 4.

References

1. **Akaike H**. 1974. A new look at the statistical model identification. IEEE Trans Automat Contr **19**:716–23.

2. **John CN**, **Stephen LX**, **Joyce Africa CW**. 2013. Is human immunodeficiency virus (HIV) stage an independent risk factor for altering the periodontal status of HIV-positive patients? A South African study. BMC Oral Health **13**:69.

3. **Khammissa R**, **Feller L**, **Altini M**, **Fatti P**, **Lemmer J**. 2012. A Comparison of Chronic Periodontitis in HIV-Seropositive Subjects and the General Population in the Ga-Rankuwa Area, South Africa. AIDS Res Treat **2012**:620962.

4. **Eren AM**, **Morrison HG**, **Lescault PJ**, **Reveillaud J**, **Vineis JH**, **Sogin ML**. 2014. Minimum entropy decomposition: Unsupervised oligotyping for sensitive partitioning of high-throughput marker gene sequences. ISME J **9**:968–979.

5. **Caporaso JG**, **Lauber CL**, **Walters WA**, **Berg-Lyons D**, **Huntley J**, **Fierer N**, **Owens SM**, **Betley J**, **Fraser L**, **Bauer M**, **Gormley N**, **Gilbert JA**, **Smith G**, **Knight R**. 2012. Ultra-high-throughput microbial community analysis on the Illumina HiSeq and MiSeq platforms. ISME J **6**:1621–4.

6. **Doyle RM**, **Alber DG**, **Jones HE**, **Harris K**, **Fitzgerald F**, **Peebles D**, **Klein N**. 2014. Term and preterm labour are associated with distinct microbial community structures in placental membranes which are independent of mode of delivery. Placenta **35**:1099–101.

7. **Morales SE**, **Holben WE**. 2009. Empirical testing of 16S rRNA gene PCR primer pairs reveals variance in target specificity and efficacy not suggested by in silico analysis. Appl Environ Microbiol **75**:2677–83.

8. **Cai L**, **Ye L**, **Tong AHY**, **Lok S**, **Zhang T**. 2013. Biased diversity metrics revealed by bacterial 16S pyrotags derived from different primer sets. PLoS One **8**:e53649.

9. **Kumar PS**, **Brooker MR**, **Dowd SE**, **Camerlengo T**. 2011. Target region selection is a critical determinant of community fingerprints generated by 16S pyrosequencing. PLoS One **6**:e20956.

10. **Chen T**, **Yu W-H**, **Izard J**, **Baranova O V**, **Lakshmanan A**, **Dewhirst FE**. 2010. The Human Oral Microbiome Database: a web accessible resource for investigating oral microbe taxonomic and genomic information. Database (Oxford) **2010**:baq013.

11. **Camacho C**, **Coulouris G**, **Avagyan V**, **Ma N**, **Papadopoulos J**, **Bealer K**, **Madden TL**. 2009. BLAST+: architecture and applications. BMC Bioinformatics **10**:421.

12. **Socransky SS**, **Haffajee AD**, **Cugini MA**, **Smith C**, **Kent RL**. 1998. Microbial complexes in subgingival plaque. J Clin Periodontol **25**:134–44.

Figure S1. Co-occurrence networks of periodontitis-associated taxa are more connected in women with periodontitis across gingivitis severities.

The co-occurrence network of periodontitis-associated taxa becomes more connected in women with periodontitis after controlling for gingivitis across the spectrum of gingivitis severity. Shown here are significant pairwise Spearman correlation coefficients ($p<0.01$, $\rho>0.4$) between periodontitis-associated OTUs in women both without **(I)** (left-hand side) and **(II)** with (right-hand side) periodontitis at all severities of gingivitis **(a)-(f)** (BoP of 1-6 respectively). Node color indicates taxonomic genus and edge weight indicates the strength of the (positive) correlation between OTUs. Node layout was determined using the Fruchterman-Reingold algorithm in qgraph v1.3.1 on the correlations in (II.f).

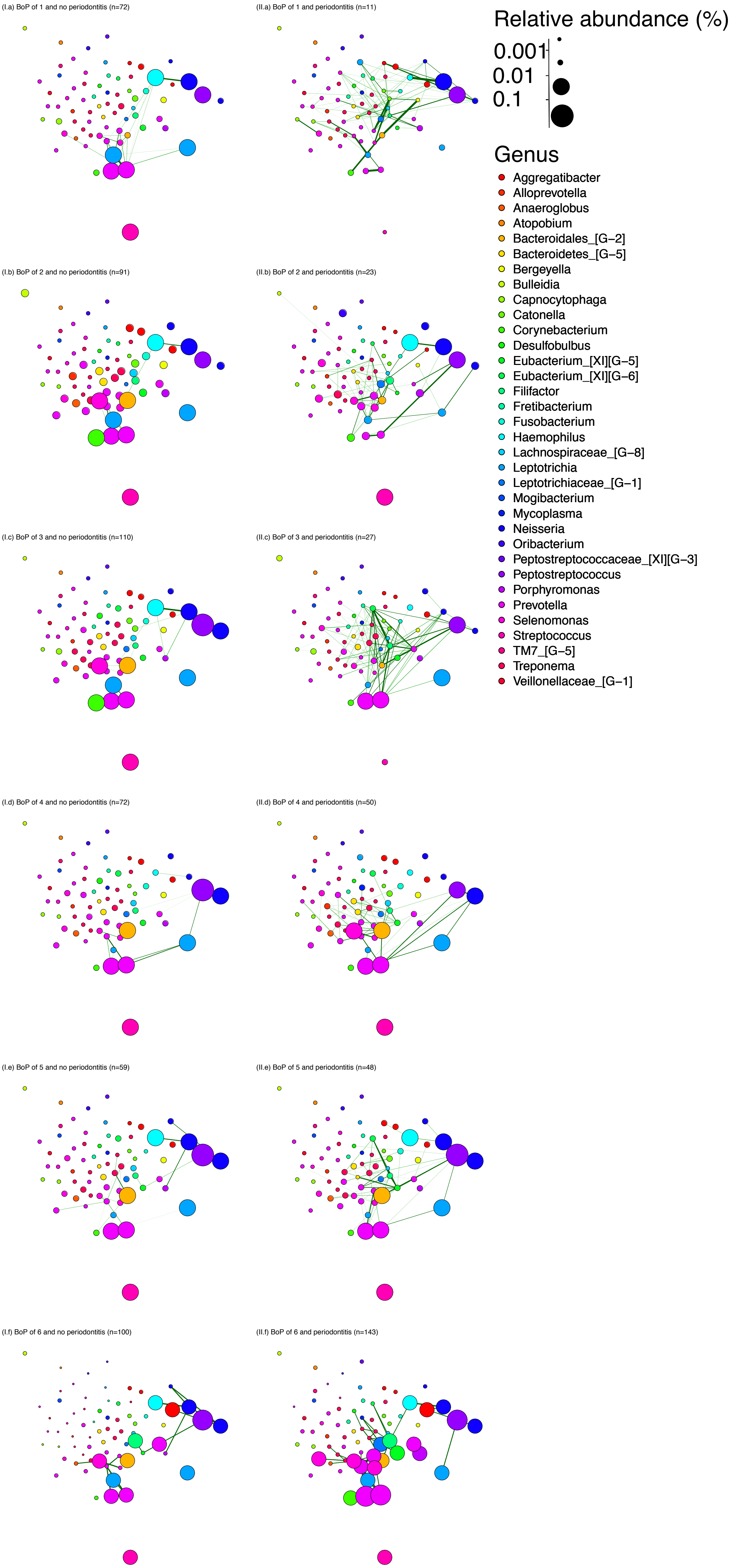

Supplement: Supplemental material [file AEM.01756-16_zam999117445so1.pdf]
